# Rapid diagnosis of COVID-19 *via* nano-biosensor-implemented biomedical utilization: a systematic review

**DOI:** 10.1039/d2ra01293f

**Published:** 2022-03-28

**Authors:** Mohammad Harun-Ur-Rashid, Tahmina Foyez, Israt Jahan, Kaushik Pal, Abu Bin Imran

**Affiliations:** Department of Chemistry, International University of Business Agriculture and Technology Dhaka 1230 Bangladesh; Department of Pharmaceutical Sciences, School of Health and Life Sciences, North South University Dhaka 1229 Bangladesh; Department of Cell Physiology, Graduate School of Medicine, Nagoya University Nagoya Japan; University Centre for Research and Development (UCRD), Department of Physics, Chandigarh University Punjab 140413 India kaushikphysics@gmail.com; Department of Chemistry, Bangladesh University of Engineering and Technology Dhaka 1000 Bangladesh abimran@chem.buet.ac.bd

## Abstract

The novel human coronavirus pandemic is one of the most significant occurrences in human civilization. The rapid proliferation and mutation of Severe Acute Respiratory Syndrome-Coronavirus 2 (SARS-CoV-2) have created an exceedingly challenging situation throughout the world's healthcare systems ranging from underdeveloped countries to super-developed countries. The disease is generally recognized as coronavirus disease 2019 (COVID-19), and it is caused by a new human CoV, which has put mankind in jeopardy. COVID-19 is death-dealing and affects people of all ages, including the elderly and middle-aged people, children, infants, persons with co-morbidities, and immunocompromised patients. Moreover, multiple SARS-CoV-2 variants have evolved as a result of genetic alteration. Some variants cause severe symptoms in patients, while others cause an unusually high infection rate, and yet others cause extremely severe symptoms as well as a high infection rate. Contrasting with a previous epidemic, COVID-19 is more contagious since the spike protein of SARS-CoV-2 demonstrates profuse affection to angiotensin-converting enzyme II (ACE2) that is copiously expressed on the surface of human lung cells. Since the estimation and tracking of viral loads are essential for determining the infection stage and recovery duration, a quick, accurate, easy, cheap, and versatile diagnostic tool is critical for managing COVID-19, as well as for outbreak control. Currently, Reverse Transcription Polymerase Chain Reaction (RT-PCR) testing is the most often utilized approach for COVID-19 diagnosis, while Computed Tomography (CT) scans of the chest are used to assess the disease's stages. However, the RT-PCR method is non-portable, tedious, and laborious, and the latter is not capable of detecting the preliminary stage of infection. In these circumstances, nano-biosensors can play an important role to deliver point-of-care diagnosis for a variety of disorders including a wide variety of viral infections rapidly, economically, precisely, and accurately. New technologies are being developed to overcome the drawbacks of the current methods. Nano-biosensors comprise bioreceptors with electrochemical, optical, or FET-based transduction for the specific detection of biomarkers. Different types of organic–inorganic nanomaterials have been incorporated for designing, fabricating, and improving the performance and analytical ability of sensors by increasing sensitivity, adsorption, and biocompatibility. The particular focus of this review is to carry out a systematic study of the status and perspectives of synthetic routes for nano-biosensors, including their background, composition, fabrication processes, and prospective applications in the diagnosis of COVID-19.

## Abstract 2

This review will focus on the rapid, selective, accurate, easy, affordable, versatile, and point-of-care diagnosis of COVID-19 using electrochemical, optical, magnetic, aptameric, and plasmonic nano-biosensors.

## Graphical abstract

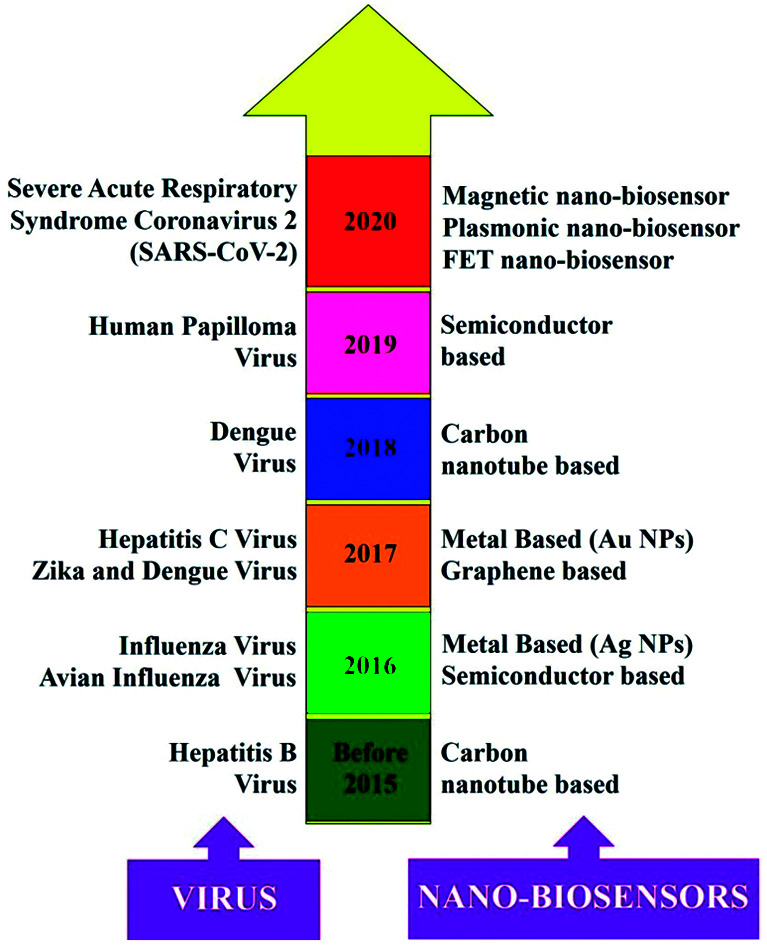

## 1 Introduction

COVID-19, which is caused by SARS-CoV-2, is a β-coronavirus and is now regarded as one of the deadliest and most highly communicable diseases.^1–3^ Infected people may or may not have symptoms but are capable of transmitting to non-infected persons.^4^ As a result, large-scale swift diagnostic testing is critical for virus diagnosis and surveillance. At present, the health care professionals associated with the diagnosis use different diagnostic tools for COVID-19 detection, such as RT-PCR, computed tomography (CT) imaging, chest X-ray radiography, serological immunoassay, and enzyme-linked immunosorbent assay (ELISA).^5^ These conventional diagnostic techniques require expert operators and highly sophisticated laboratory facilities.^6^ In addition to complex laboratory setups, the gaps between sampling, diagnosis, and the decrease in viral load due to responsive natural immune system have inspired scientists and technologists to further work on the development of rapid and reliable diagnostic methods for the massive screening process of COVID-19 detection.^7^ The identification of viruses and the confirmation of diseases by nano-biosensors have more advantages over the conventional diagnostic tools in terms of sensitivity, response time, reliability, and portability.^8^ Nano-biosensors are capable of detecting samples as low as 10^−18^ M,^9^ are highly sensitive,^10^ have the potential to overcome the drawbacks associated with false negatives and positives rendered by RT-PCR tests,^11^ and are suitable to be utilized as very handy devices for easy and effortless applications around point-of-care centers.

Nano-biosensors are fabricated using biological molecules. Different types of biological molecules, including proteins, antibodies, aptamers, enzymes, nucleic acids, and cells are usually immobilized by chemisorption and/or physisorption on the surface of the transducer. The biochemical interactions among antibodies, antigen, and protein and the hybridization of nucleic acid occurring on the surface of the transducer are measured by electrochemical, optical, piezoelectric, magnetic, mechanical, and thermal changes. Nanoparticles are employed to enhance the performance of biosensors by elevating the ultrasensitive spotting and identification of biomarkers present in the samples. They have been playing vital roles in developing the nano-biosensors for diagnostic and detection platforms as they offer excellent electronic and optical properties, high chemical reactivity, better stability, greater surface, good adsorption, robustness, biocompatibility, quick and enhanced-sensitive diagnosis with minimal sample (patient's nose swab, throat swab, saliva sputum, and blood) volume, which are important for COVID-19 detection. Different types of nanomaterials such as lanthanide-doped polystyrene nanoparticles, gold-based nanostructures, iron oxide, magnetic particles, carbon black, and graphene nanoparticles have been utilized for the development of electrochemical, optical, and magnetic nano-biosensors for COVID-19 prognosis.^12,13^

This review covers diverse nano-biosensors, including electrochemical, optical, magnetic, aptameric, and plasmonic sensors for the spotting of SARS-CoV-2 and for COVID-19 prognosis. A brief representation of smart nano-biosensors such as wearable and stretchable nano-biosensors operated by smartphone interfaced applications to assist rapid detection of SARS-CoV-2 and COVID-19 diagnosis is also be included in this review. The advantages, problems, prospects, and challenges associated with the fabrication, applications, and commercialization of nano-biosensors are also discussed.

## 2 Nano-biosensors as point-of-care diagnostic tools

**Fig. 1:**
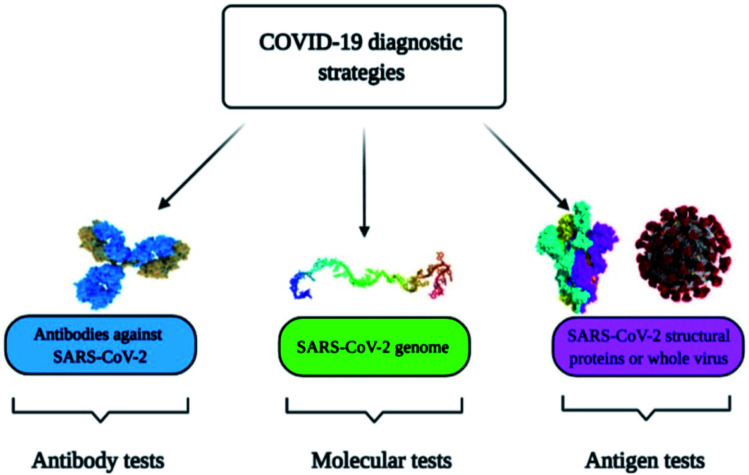
Commonly used methods for the spotting of SARS-CoV-2 and COVID-19 prognosis. Reprinted (adapted) with permission from (ref. 15) copyright 2021, MDPI.

**Fig. 2:**
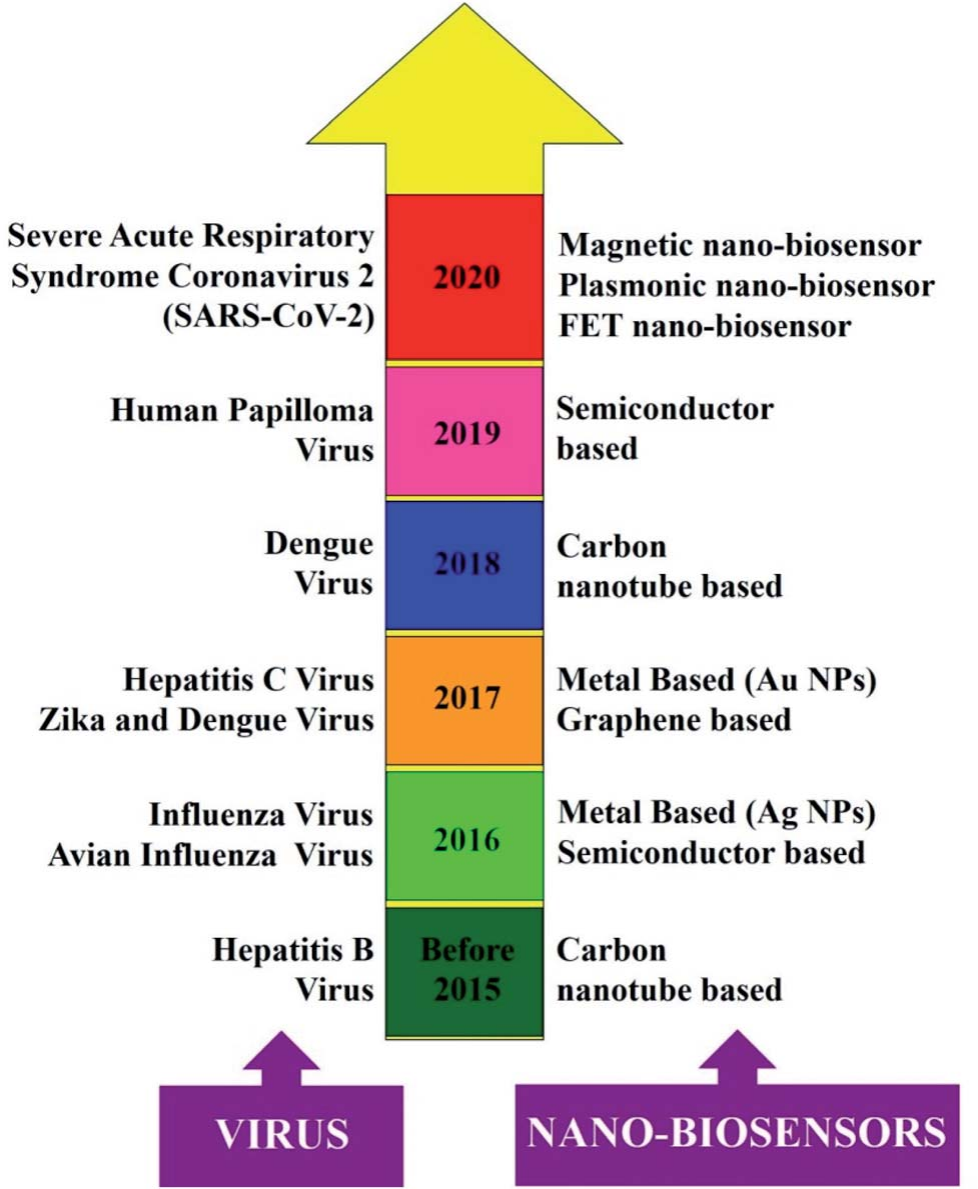
Chronology of the different nano-biosensors developed for the detection of other virus strains. Reprinted (adapted) with permission from (ref. 20) copyright 2021, Wiley.

Diagnostic tools for point-of-care spotting of SARS-CoV-2 and COVID-19 prognosis have found applications in many developing and underdeveloped countries. In association with point-of-care identifications, immunoassay methods have received more recognition than molecular diagnostic-based tools due to their better control and the generation of fewer false-positive cases. Moreover, the *in situ* detection process could be a straightforward, expeditious, and economical method that does not require any additional skills for instrument operation. Nano-biosensors are pertinent tools for the beside-testing of COVID-19 to offer care near to the patient.^14^ Presently, the commonly used diagnostic tools for the spotting of SARS-CoV-2 are antibody, molecular, and antigen-based (Fig. 1).^15–19^ The advantages and disadvantages of several diagnostic procedures used in detecting SARS-CoV-2 are summarized in Table 1. For the last several years, there has been tremendous development in nano-biosensor fabrication for detecting different virus strains. Fig. 2 represents the chronology of the developed nano-biosensors for viral disease diagnosis.

**Table 1:** The advantages and disadvantages of several diagnostic procedures used in the detection of SARS-CoV-2

Sl#	Test name	Advantages	Disadvantages
1	Molecular test: Nucleic acid amplification test (NAAT), RT-PCR, Loop-mediated isothermal amplification (LAMP)	Gold standard diagnosis test, highly accurate with greater specificity and sensitivity	Require laboratory facilities, trained personnel, sophisticated sample collection, preparation, and storage, slow (3–6 h), complicated, expensive, false negative result
2	Antigen test	Fast (15–30 min), inexpensive, simple, offers quick detection and screening	Potential for false positives, average sensitivity, poor accuracy, qualitative test only
3	Antibody test: ELISA	Simple, specific, sensitive, efficient, safe, and eco-friendly	Labor-intensive, expensive antibody and culture media required, false negative result, difficulties in transport and storage
4	Nano-biosensors	High sensitivity and selectivity, fast, suitable for automation, label-free detection, better stability and reproducibility, low detection limit, wide range of response limit	Susceptible to sample matrix, inferior shelf life, need extensive research for commercialization and mass production

### 2.1 Electrochemical nano-biosensors

**Fig. 3:**
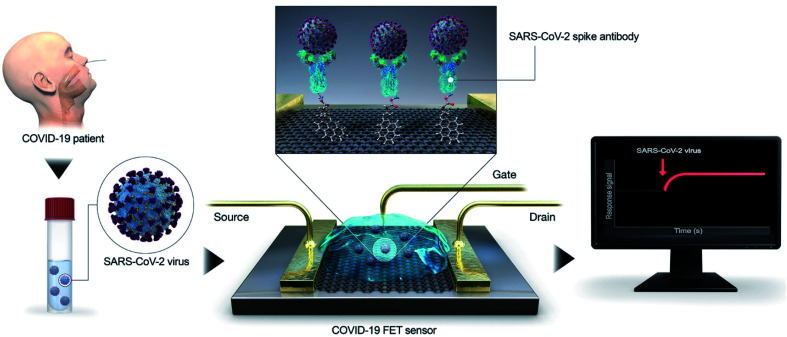
Simple illustration of an immuno-biosensor manufactured by coating the graphene sheet of a FET with a clearly defined protein found in coronavirus spikes. Reproduced (adapted) from (ref. 30) copyright 2020, ACS.

Recently, transistor-oriented tools have been considered as effective platforms for the cost-effective and rapid sensing of various species. In this context, organic transistors have found potential applications due to their ability to conjugate with biological systems and detect SARS-CoV-2 (COVID-19).^21–29^ To enhance the detection limit for solving the aforementioned problem, Seo's group fabricated a graphene oriented nano-biosensor to spot SARS-CoV-2 and for COVID-19 prognosis (illustrated in Fig. 3) for the SARS-CoV-2 virus identification platform. The proposed immunosensor was manufactured by coating the graphene sheets of a field-effect transistor (FET) with a clearly defined protein found in coronavirus spikes.^30^

**Fig. 4:**
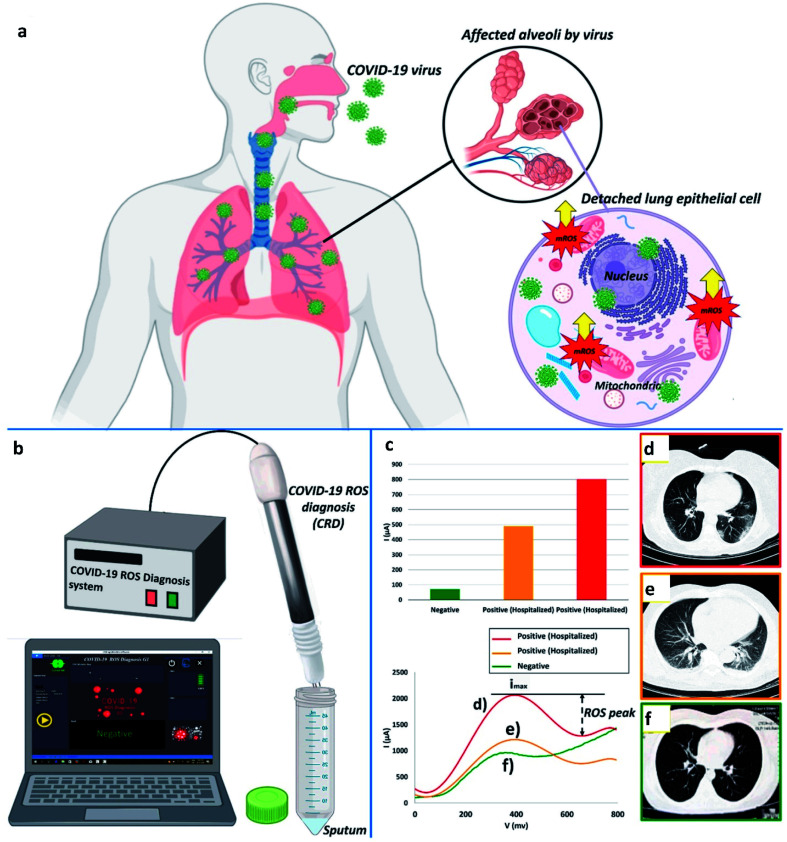
(a) Typical representation of ROS, (b) device for ROS detection, (c) ROS detected in different patients, and (d), (e) contrasted with a verified sample. (f) Red, orange, and green represent 800 μA, 490 μA, and ∼71 μA, respectively. Images produced by lung CT scanning (d, e, and f). Reprinted (adapted) with permission from (ref. 10) copyright 2021, Elsevier.

Fathi-Hafshejani and colleagues reported the utilization of FET-based two-dimensional (2D) semiconductor materials in *in vitro*^31^ sensing applications for the fast detection and identification of the coronavirus that causes COVID-19. Song's group developed an electrochemical nano-biosensor based on newly designed peptides and electropolymerized polyaniline (PANI) nanowires for COVID-19 prognosis with enhanced antifouling capabilities.^32^ Taking the reactive oxygen species (ROS) level into consideration as one of the key side effects reported for SARS-CoV-2, Miripour and colleagues have introduced a simple electrochemical sensor (illustrated in Fig. 4) to selectively detect SARS-CoV-2 and for COVID-19 prognosis.^10^

**Fig. 5:**
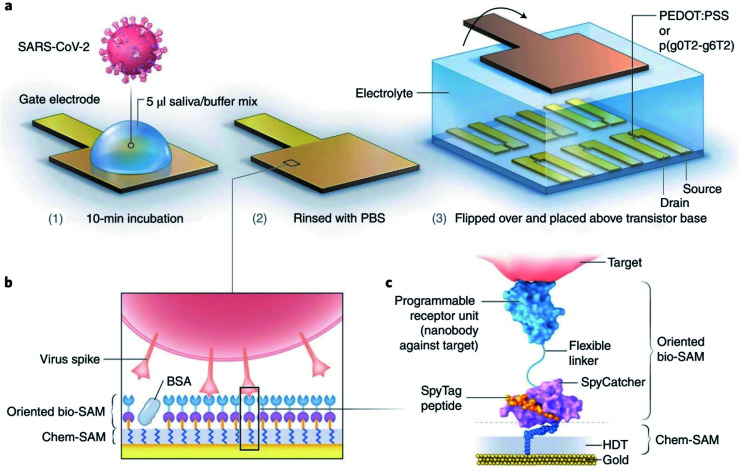
(a) Functionalities of sensors, (b) different layers for operation purposes, and (c) the molecular structure of proposed nano-biosensors. Reprinted (adapted) with permission from (ref. 37) copyright 2021, Springer Nature.

Several reports have already been published on smartphone-based electrochemical impedance spectroscopy (EIS) oriented biosensors comprised of diverse nanomaterials including nanodendroids, nanoparticles, and graphene oxide (GO) nanocomposites for COVID-19 detection.^33,34^ Shan's group has prepared a nano-biosensor-based breath device composed of gold nanoparticles (AuNPs) and organic ligands with multiplexed detection capabilities to spot SARS-CoV-2 by analyzing the breath of suspected patients.^35^ Significant progress has been realized in developing organic electrochemical transistors (OECTs) that transduce and amplify biological changes into an electrical signal.^36^ Guo's group reported OECTs with a modular architecture to spot coronavirus spike proteins present in samples collected from patients.^37^ The sensor has been illustrated in Fig. 5. Several types of OECT-based nano-biosensors are effectively capable of spotting the protein present in corona virus spikes.^38,39^

**Fig. 6:**
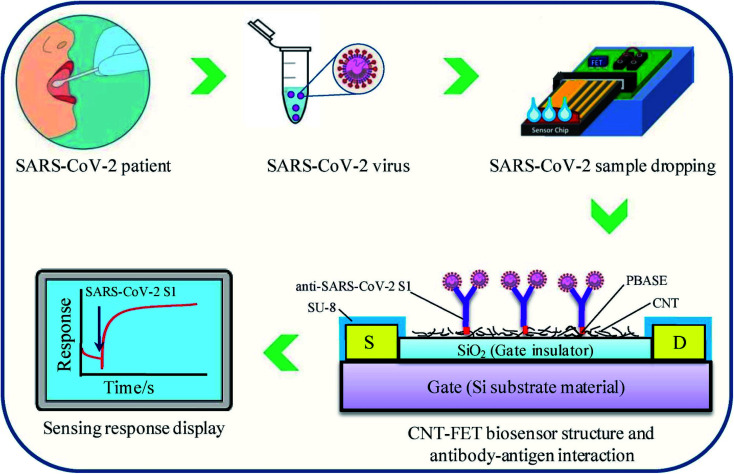
CNT-FET-based nano-biosensor and SARS-CoV-2 S1 detection process. Reprinted (adapted) with permission from (ref. 40) copyright 2022, Elsevier.

Zamzami's group developed an electrochemical nano-biosensor composed of carbon nanotube FET (CNT-FET) for detecting the SARS-CoV-2 spike protein (S1) in a patient's saliva.^40^ The detection is rapid (2–3 min), accurate, cost-effective, simple, and quantitative. CNT printing was used to create a biosensor on a Si/SiO_2_ surface with anti-SARS-CoV-2 S1 immobilization. The SARS-CoV-2 S1 antibody is immobilized on the surface of carbon nanotubes by non-covalent bonding with a linker, 1-pyrenebutanoic acid succinimidyl ester. To simulate a SARS-CoV-2-positive saliva sample, 1 μL SARS-CoV-2 S1 antigen was mixed with 99 μL saliva from a non-infected person and incubated at 37 °C for 30 minutes. During the process, the concentration of the SARS-CoV-2 S1 antigen in modified human saliva was maintained at 0.1, 1, 10, 100, 1000, 2000, 3000, 4000 and 5000 fg mL^−1^. Instead of a clinical SARS-CoV-2 positive sample, this enriched saliva solution was employed as a test sample. For reading purposes, 1.0 μL of the artificial SARS-CoV-2-positive saliva sample was added to the anti-SARS-CoV-2 S1 embedded biosensor, and the electrical indication was recorded for 1 to 2 min. Commercially available SARS-CoV-2 S1 antigen was applied to evaluate the electrical signals of the sensor. The proposed sensor can effectively detect the SARS-CoV-2 S1 antigen present in the 10 mM buffer at pH 6.0 at concentrations from 0.1 fg mL^−1^ to 5.0 pg mL^−1^, with the limit of detection (LOD) of 4.12 fg mL^−1^. The specificity of the sensor was explored by utilizing SARS-CoV-2 S1 as the target species, with SARS-CoV-1 S1 and MERS-CoV S1 antigens as non-target species in pH 6.0 medium. The biosensor is capable of showing high specificity in the case of SARS-CoV-2 S1 antigen identification. The structure of carbon nanotube field-effect transistor (CNT-FET)-based nano-biosensor and its detection process are illustrated in Fig. 6.

### 2.2 Optical nano-biosensors

**Fig. 7:**
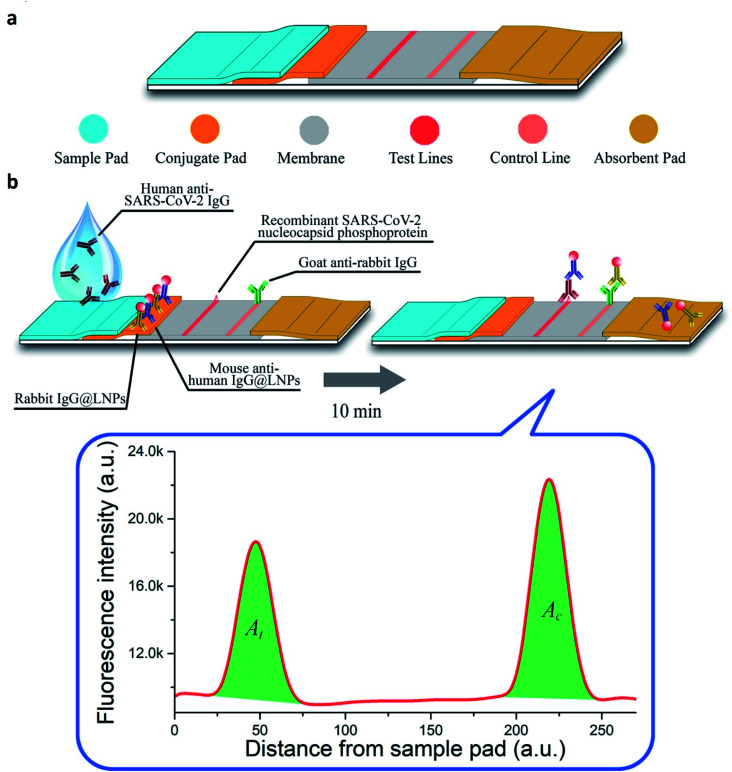
(a) Structural and (b) functional representation of LFIA sensors. Reprinted (adapted) with permission from (ref. 48) copyright 2020, ACS.

In optical nano-biosensors, the changes associated with the interactions between the bioreceptor and analyte are elucidated by fluorescence, luminescence, surface plasmon, plasmon-enhanced fluorescence, surface-enhancing Raman scattering, colorimetry, reflectance, and absorbance. These correspond to the concentration of the analyte and types of nanomaterial.^41–44^ Peng and colleagues have developed a colloidal AuNPs-based chemiluminescence immunoassay for the quick and precise detection of immunoglobulin-M (IgM) and immunoglobulin-G (IgG) of SARS-CoV-2.^45^ Diao's group has evaluated the significance of fluorescence signals for spotting SARS-CoV-2 protein present in patient samples in 10 min.^46^ Single-walled CNT-based nano-biosensors can also detect the presence of protein through the optical readout.^47^ Murugan's group has reported mobile plasmonic fiber-optic absorbance biosensor (P-FAB) sensors.^9^ Chen and coworkers have reported a lateral flow immunoassay (LFIA), which is rapid and doped with lanthanide-modified polystyrene nanoparticles for detecting IgG antibodies in patient serum against SARV-CoV-2 (illustrated in Fig. 7).^48^

**Fig. 8:**
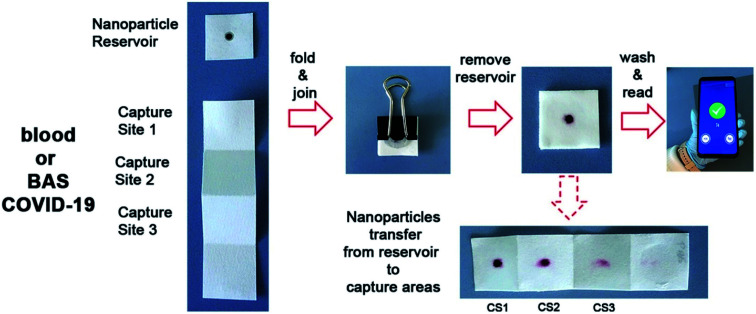
The procedure for the detection of IL-6 present in blood samples of patients by a paper-based biosensor with the help of a smartphone application. Reprinted (adapted) with permission from (ref. 49) copyright 2021, Elsevier.

A biosensor, consisting of a paper immunosensor capable of generating intense colorimetric signals and communicating with a smartphone aimed at detecting severe cases of COVID-19, has been proposed by Adrover-Jaume and colleagues.^49^ They have proposed the sensor as a diagnostic biomarker for COVID-19 to detect the ultra-low concentrations of interleukin 6 (IL-6) in blood samples of patients. The detection procedure (Fig. 8) is very simple to conduct, and it can detect cytokines in the blood and respiratory samples of patients.

Hadi and Khurshid have developed an optical fiber-based nano-biosensor for COVID-19 detection.^50^ A novel U-shaped optical fiber (POF)-based sensing probe has been constructed by utilizing polymethylmethacrylate (PMMA) as a core with the refractive index (RI) of 1.49. The core was coated with a fluorinated polymer with an RI of 1.41. Variations in the probes can be minimized by carefully selecting probes with RI sensitivity, which are evaluated by exposing the probe to sucrose solutions of varying concentrations. The AuNPs were synthesized by a citrate-mediated reduction process and by immobilization on the surface of a U-bent optical probe. Later, anti-N protein monoclonal antibodies were conjugated to achieve greater antibody binding affinity, which was evaluated by the ELISA method. The selectivity of the probe was ensured by treating it with bovine serum albumin (BSA). The resultant biofunctionalized probes were applied in SARS-CoV-2 detection from saliva samples. The sensor is even capable of detecting Omicron, the latest variant of SARS-CoV-2. The manipulation of the signal received by the sensor is accomplished by several techniques such as wavelength division multiplexing, intensity modulation, frequency multiplexing, and so on. Among these techniques, the intensity modulation is the fastest and is well balanced. For testing purposes, the sample is collected from the nasopharyngeal/oropharyngeal area by a specific POF U-shaped probe with one terminal while the other terminal is interlinked to a photodetector to obtain the signal for further processing. The experimental results claim that the detection would be accurate in the case of POF with a reduced diameter. The real-time detection of SARS-CoV-2 and its variants can be very accurately, efficiently, and economically done within 15 min. The inventors expect that the technique will make the detection process quicker and more cost-effective and suitable for screening patients living in remote locations where there is a lack of clinical laboratories due to scarcity of funding and geographic limitations.

### 2.3 Magnetic nano-biosensors

**Fig. 9:**
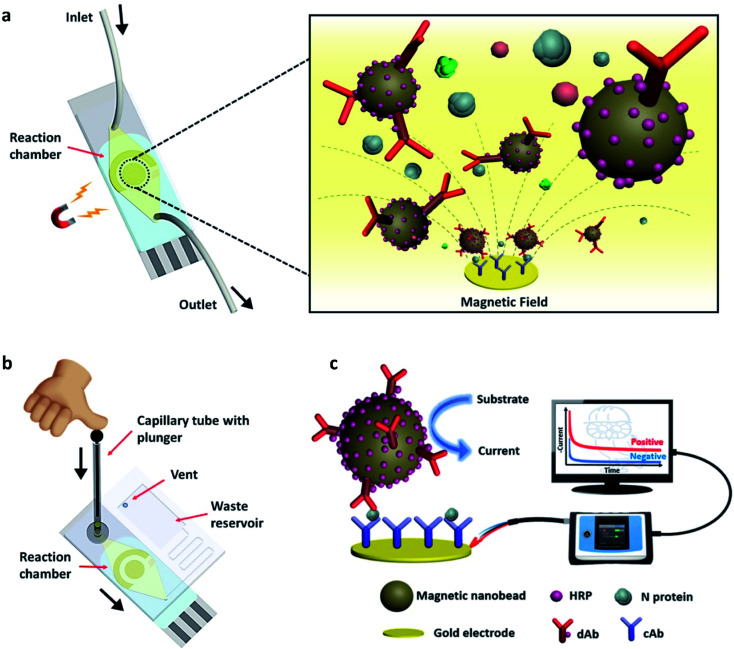
(a) Simple illustration of the surface of the nano-biosensor, (b) test procedure, and (c) the complete setup of the device. In the figure, HRP, dAb, and N protein stand for horseradish peroxidase, detection antibody, and nucleocapsid proteins, respectively. Reprinted (adapted) with permission from (ref. 51) copyright 2021, ACS.

The advantage of a magnetic nano-biosensor over an electrochemical or optical nano-biosensor is that it produces less background noise due to the non-magnetic nature of the biological environment. Li and Lillehoj have demonstrated a novel immunosensor composed of magnetic nanoparticles.^51^ This unique immunosensor works (illustrated in Fig. 9) *via* immunomagnetic signal amplification and can detect the virus protein with reproducibility and sensitivity.

**Fig. 10:**
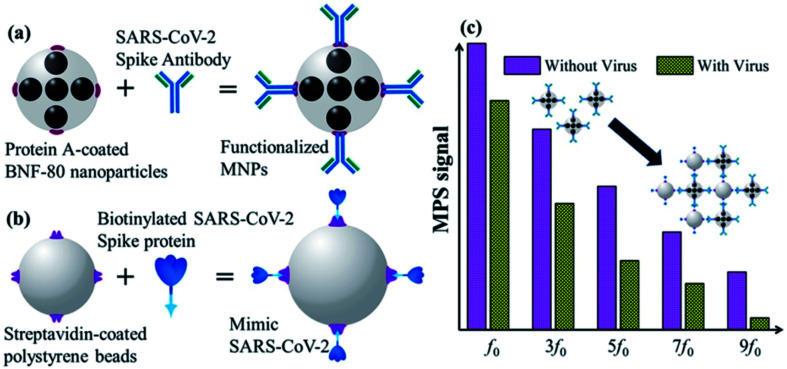
Schematic illustration of functionalized magnetic nanoparticles (a), mimetic SARS-CoV-2 (b), and MPS signals (c). Reprinted (adapted) with permission from (ref. 54) copyright 2021, ACS.

A unique and quick serological magnetic immunodetection (MID) point-of-care assay has been manufactured with high sensitivity of 97% and specificity of 92%.^52^ Pietschmann's group has demonstrated the application of portable magnetic particle spectroscopy (MPS) surface-based Magnetic Immuno-Detection for SARS-CoV-2 specific antibodies.^53^ Huergo and colleagues have developed a facile chromogenic magnetic bead-based immunoassay (illustrated in Fig. 10) that allows the quick, cost-effective, and quantitative identification of SARS-CoV-2 in patient samples.^54^

**Fig. 11:**
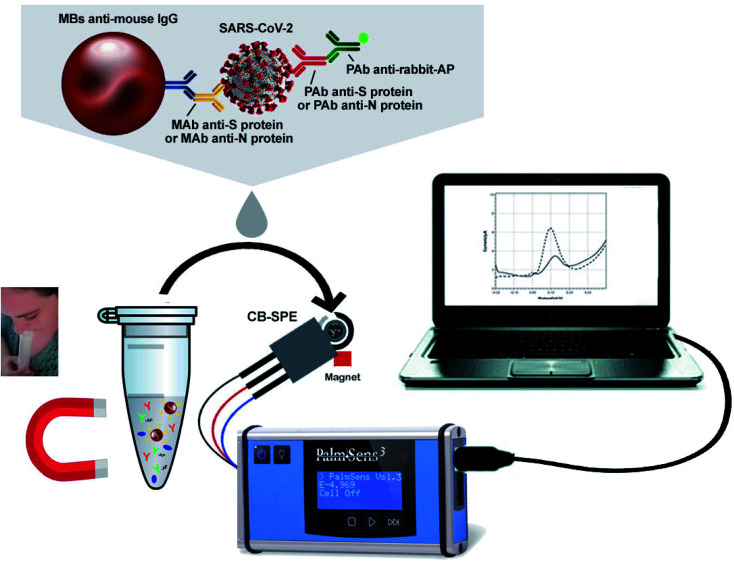
Typical illustration of the structure and operation of magnetic nanoparticles-based nano-biosensors. Reprinted (adapted) with permission from (ref. 55) copyright 2021, Elsevier.

Fabiani's group has developed a nano-biosensor comprised of magnetic nanoparticles and carbon black-composed screen-printed electrodes (SPEs) for detecting SARS-CoV-2 (illustrated in Fig. 11) from the saliva of patients.^55^ The biosensing procedure is conducted for either nucleocapsid (N) or spike (S) protein by utilizing magnetic beads that act as the supporting material for an immunological chain and a secondary antibody alkaline phosphatase as the immunological label. The enzymatic by-product, 1-naphthol, is detected with the help of screen-printed electrodes fortified with carbon black nanoparticles. The analytical characteristics of the sensor were explored by analyzing the raw saliva of the S protein solution and N protein solution in a buffer. The detection limit of the reported magnetic nano-biosensor for the S protein is 19 ng mL^−1^, and 8 ng mL^−1^ for the N protein. The potential of the sensor was evaluated by using the cultured virus in saliva and biosafety level 3, contrasting the data collected from the real-time PCR results of the nasopharyngeal swab sample test. Experimental data demonstrated that the device is highly sensitive with a lower detection limit and is rapid (30 min for detection), handy, and highly promising for quick commercialization.

### 2.4 Aptameric nano-biosensors

**Fig. 12:**
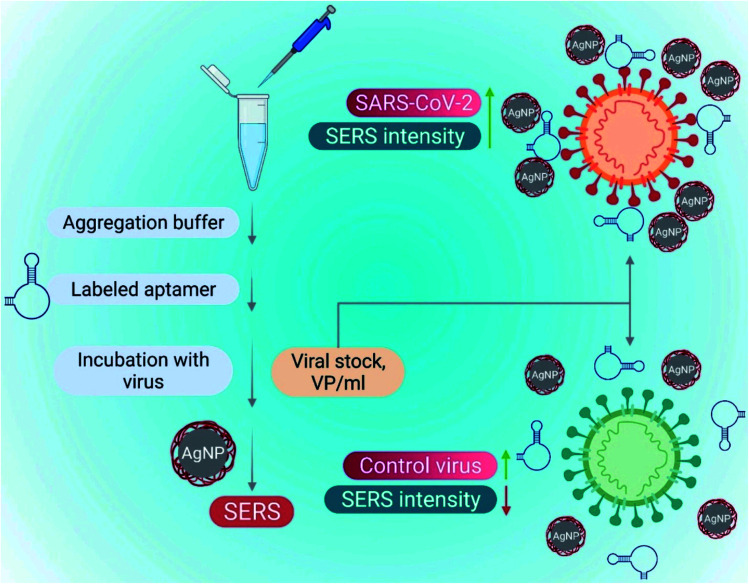
SARS-CoV-2 detection utilizing a surface-enhanced Raman spectroscopy (SERS)-based aptasensor constructed on silver nanoparticles (AgNPs). Reprinted (adapted) with permission from (ref. 58) copyright 2022, Elsevier.

The introduction of aptamers can enhance the sensitivity and selectivity of nano-biosensors. Aptamers are short single-stranded (25–90 bases length) oligonucleotides. The 3D spatial arrangements of aptamers play a significant role in detecting viral components due to their high combining ability. Nano-biosensors conjugated with the aptamers have an excellent advantage for detecting COVID-19. Aptameric nano-biosensors can perform real-time analysis and act against multiple targets. Some of the DNA aptamers, such as MSA1 and MSA5 possess high binding affinity (few nM range) to the spike protein (S1) subunit of the SARS-CoV-2.^56^ Applying the machine learning algorithms, Song's group has isolated CoV2-RBD-1C and CoV2-RBD-4C aptamers, which specifically target the receptor-binding domain (RBD) of the SARS-CoV-2 spike protein with a strong attraction to angiotensin-converting enzyme II (5.8 nM and 19.9 nM respectively).^57^ The aptameric sensor-based diagnosis procedure has been illustrated in Fig. 12.

**Fig. 13:**
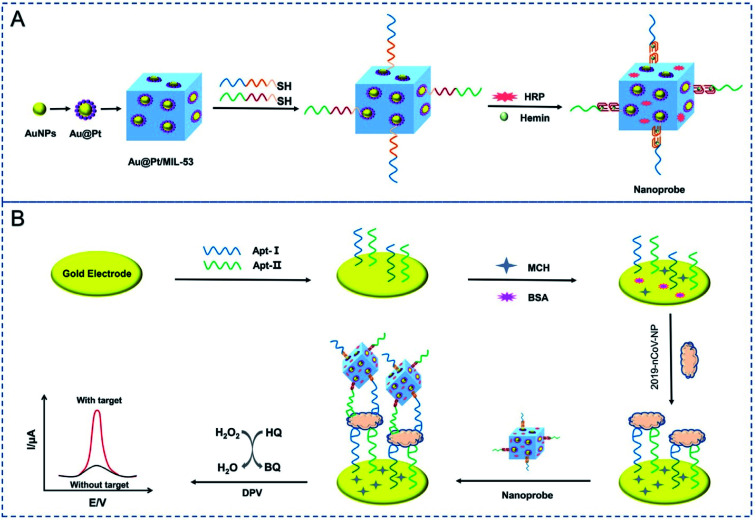
Schematic depiction of the fabrication processes for the nanoprobes (A) and the electrochemical aptasensor for the capture and detection of 2019-nCov-NP (B). Reprinted (adapted) with permission from (ref. 59) copyright 2021, Elsevier.

Tian and coworkers constructed a dual-aptameric nano-biosensor composed of an organo-metallic substrate reinforced with AuNPs and platinum nanoparticles and enzymes for SARS-CoV-2 nucleocapsid protein (2019-nCoV-NP) detection through the co-catalysis of the nanomaterials, G-quadruplex DNAzyme, and HRP.^59^ Initially, the two aptamers (N48 and N61) modified with thiol were immobilized on the surface of a gold electrode (GE) to capture the biomarker 2019-nCoV-NP. Subsequently, metal nanocomposites composed of Au@Pt/MIL-53 (Al) were fortified with HRP and hemin/G-quadruplex DNAzyme as a signal nanoprobe. Eventually, a highly reliable, selective, and sensitive aptameric nano-biosensor was manufactured on the GE surface for the early diagnosis of COVID-19 with a very low detection limit of 8.33 pg mL^−1^. For evaluating the selectivity of this aptameric sensor, several proteins, including MPT64, 2019-nCov-NP, Her2, and cTnI, and a blank sample without any presence of protein were analyzed. The experimental results showed that the sensor can detect 2019-nCov-NP, which complies with the high selectivity of the aptameric nano-biosensor. The reproducibility of the results was explored *via* three different processes and each one was conducted thrice. The results confirmed the good reproducibility of the sensor with the standard deviation of 2.6%, 4.3%, and 5.0% for 0.1, 1 and, 10 ng mL^−1^ of 2019-nCov-NP detection, respectively. The fabrication of the sensor and the detection mechanism are demonstrated in Fig. 13.

Abrego-Martinez and coworkers developed a facile, rapid, sensitive aptameric nano-biosensor constructed on a screen-printed carbon electrode *via* the immobilization of the aptasensor on AuNPs for SARS-CoV-2 detection. The detection mechanism depends on the binding affinity of the aptamer towards the target receptor-binding domain (RBD) present in SARS-CoV-2's spike protein (S-protein).^60^ The aptameric nano-biosensor exhibits an outstanding detection performance of 1.30 pM (66 pg mL^−1^) SARS-CoV-2 S-protein, even though 40 minutes incubation time is required, which is lower than the standard diagnostic procedures. The LODp of PCR for the same analyte is higher than the reported sensor. The selectivity exploration of the sensor demonstrates that it is active to the spike proteins of both SARS-CoV-2 and SARS-CoV. It should be noted that the sensor is more selective towards the spike protein of SARS-CoV-2 than SARS-CoV, since the former is more positively charged. In addition, the sensor has been specifically developed for use as a tool for the portable and rapid detection of COVID-19 by connecting with a handheld potentiostat linked to a smartphone. Since the fabrication method is straightforward, the sensor can be reproduced in any industrial facility for commercial mass production.

### 2.5 Plasmonic nano-biosensor

Plasmons are produced as a consequence of electromagnetic light waves interacting with the free electrons available on the surface of metal nanoparticles. A plasmonic metallic film is comprised of a 50 nm metal nanoparticle coating on the prism. The specimen is composed of the running buffer for commercially available plasmonic sensors. The cautious application of commercially available plasmonic nano-biosensors could be effective for real-time and label-free detection with greater reproducibility and reusability of the sensor chips. Das and colleagues have proposed a model of a sandwich-type plasmonic nano-biosensor comprised of AuNPs to diagnose COVID-19 *via* SARS-CoV-2 spike protein detection.^61^

**Fig. 14:**
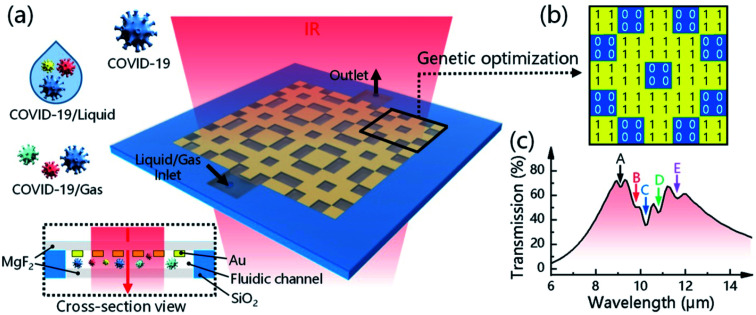
Plasmonic biosensors for detecting and identifying COVID-19. (a) Device configuration and sensing mechanism of the 3D plasmonic sensor. (b) A genetic algorithm (GA) program is used to optimize the metal structure, where “1” means nano-metal and “0” means no nano-metal. (c) The resonance position of the metal structure is designed to overlap the fingerprint vibration signals of the virus molecule, allowing the simultaneous enhancement and detection of COVID-19-induced absorption changes. Reprinted (adapted) with permission from (ref. 62) copyright 2021, ACS.

Li's group has reported a rapid, extremely sensitive, and multi-faceted plasmonic nano-biosensor based on surface-enhanced infrared absorption for on-spot COVID-19 detection.^62^ An intelligent genetic algorithm program has been utilized for the spontaneous design and rapid optimization of the sensor to improve the overall detection performance. The sensor is highly sensitive for detecting COVID-19 quantitatively (1.66%/nm). It is worth mentioning that the sensor might be an ideal tool for detecting the variants of viruses due to the presence of distinctive infrared fingerprint recognition features. The plasmonic nano-biosensor and the COVID-19 detection are illustrated in Fig. 14.

**Fig. 15:**
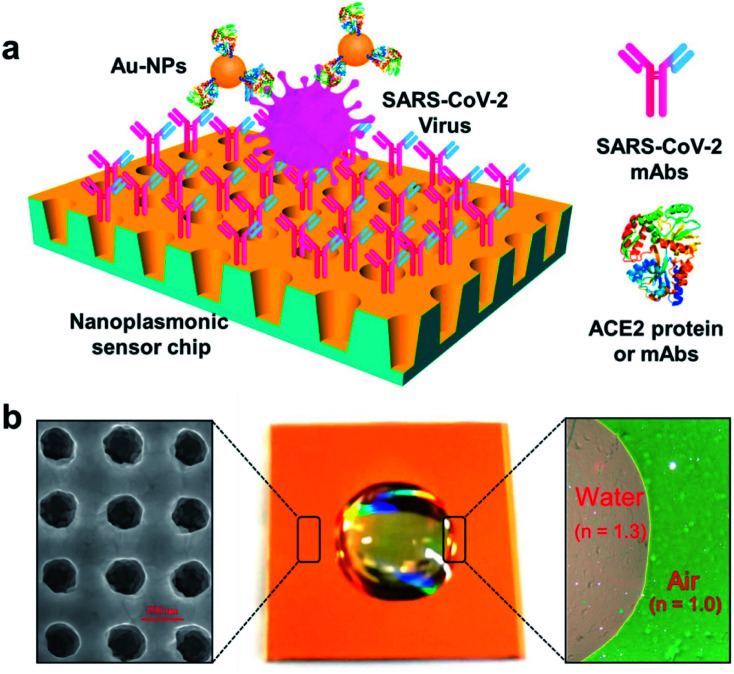
Label-free detection of the SARS-CoV-2 pseudovirus with a nanoplasmonic sensor. (a) Schematic diagram of the nanoplasmonic resonance sensor for the determination of the SARS-CoV-2 pseudovirus concentration. (b) A photograph (middle) showing one piece of the Au nanocup array chip with a drop of water on top. Scanning electron microscopy image (left) showing the replicated nanocup array. Transmission microscopy image (right) showing that air and water on the device surface exhibit different colors; green and far-red pink, respectively. Reprinted (adapted) with permission from (ref. 63) copyright 2021, Elsevier.

Some researchers have fabricated various nano-biosensors based on surface plasmon resonance for COVID-19 detection. Liu and coworkers have employed a gold nanocup arrangement to determine the spike protein of SARS-CoV-2.^63^ Concentrations as low as 370 vp mL^−1^ can be detected in one step in 15 min, and the concentration of viral particles can be quantitatively analyzed in the range of 0 to 10^7^ vp mL^−1^ linearly. The test results can be obtained from a generic microplate reader and analyzed from a smartphone-connected device. The versatility of demonstration of the test results suggests that the manufactured sensor can be rapidly acquired under both regular clinical setups as well as resource-limited environments. The detection and quantitative analysis of SARS-CoV-2 have been illustrated in Fig. 15.

There are many notable examples of applications of plasmonic nano-biosensors for the detection of COVID-19 and other diseases.^64^ The representative plasmonic nano-biosensors and their performances in diagnosing COVID-19 are condensed in Table 2.

**Table 2:** A summary of typical plasmonic nano-biosensors and their performance in COVID-19 detection

Sl#	Nanomaterial	Target	Detection limit	Reference
1	Au nanospike	S Protein	0.08 ng mL^−1^	65
2	Au nanocup	S Protein	370 vp mL^−1^	63
3	Au nanoisland	SARS-CoV-2	0.22 pM	44,66
4	Au nanorod	S Protein	111.11 deg RIU^−1^	61
5	AuNPs	N gene	0.18 ng uL^−1^	67
6	AuNPs	S Protein	4.2 fM	68
7	Au nanohole	S Protein	—	69
8	AuNPs	RNA	160 fM	70
9	AuNPs	RNA	3.2 gene uL^−1^	71

Although there has been significant development regarding the plasmonic nano-biosensor, there is still a huge requirement to improve sensing devices and commercialization. To date, the detection process of plasmonic biosensors is limited to serum but it should be more versatile for other types of samples, including whole blood, saliva, sweat, and urine collected from patients. The plasmonic nano-biosensor could be more effective by trapping and processing light on the chip, and maneuvering individual chemical samples on small chip structures to magnify the role of the chip; it may additionally upgrade the development of plasmon miniaturization.^72–77^ With the quick development of optical technology, the tools with sophisticated functions are promising to be more workable and portable for real-time sample analysis.^78^ There are currently no fully functioning diagnostic tools in the market based on plasmonic sensors. The application of metamaterials in the design and development of plasmonic nano-biosensor may create an opportunity to enhance the detection ability and commercialization process by reducing the manufacturing cost. The evolution of new materials may improve detection methods. For example, TiN is replacing AuNPs in plasmonic detection technology since the former is chemically stable. More importantly, TiN is available and cheap, which ensures its prospects for commercial production and application in biosensing technology.^79,80^

### 2.6 Nano-biosensors for the detection of other viral strains

The diagnosis of viral infectious disease can be conducted using nano-biosensors developed by modifying CNT-based electrodes composed of metallic nanoparticles.^81^ Wiriyachaiporn and colleagues have employed carbon nanotag-based lateral flow assay to diagnose the different strains of the influenza A virus by maintaining optimum conditions.^82^ In this nano-biosensor, carbon nanoparticles perform as receptors in the shape of nano strings. Another research group has manufactured nano-biosensors containing CNTs and 4,40′-diaminoazobenzene to diagnose the hepatitis B virus (HBV) using the electrochemical method.^83^ A cost-effective and portable graphene-based nano-biosensor has been reported for identifying the Zika virus with the help of a highly specific immobilized monoclonal antibody by Afsagi's group.^84^ This sensor's working principle is field-effect biosensing-dependent, enabling the quantitative and real-time diagnosis of native Zika viral antigens. Navakul's group has developed a novel method for the rapid detection, classification, and antibody screening of the dengue virus based on electrochemical impedance spectroscopy (EIS) using graphene reinforced polymer-coated gold electrode nano-biosensors.^85^ Laderman and colleagues have prepared a rapid, selective, and sensitive nano-biosensor from AuNPs for detecting the herpes simplex virus type 2 (HSV-2) by applying a lateral-flow immunochromatographic assay.^86^ By taking advantage of the outstanding physicochemical and optical characteristics of AuNPs, a nano-biosensor has been developed for the quantitative detection of the hepatitis C virus (HCV).^87^ Among the metallic nanoparticles, AgNPs are the most widely utilized in the diagnosis of pathogens in biological systems. Zou's group has fabricated a label-free fluorescence sensor comprised of DNA-stabilized Ag nanoclusters for determining two human immunodeficiency virus oligonucleotides (HIV DNAs) simultaneously in the early stage of infection.^88^ Applying the electrochemical technique, Sepunaru and colleagues prepared a nano-biosensor utilizing AgNPs for the quick identification of the influenza virus in real-time.^89^ Avian influenza can be detected efficiently and sensitively by using an electrochemical DNA nano-biosensor based on ZnO nanoparticles modified graphene.^90^ Nano-biosensors for detecting Hepatitis, HIV, Ebola, Influenza, Herpes, and Human papillomavirus are summarized in Table 3.

**Table 3:** A summary of nano-biosensors designed to detect Hepatitis, HIV, Ebola, Influenza, Herpes, and Human papillomavirus

Viruses	Nanoparticles	LOD	Detection range	Ref.
**Hepatitis**				
HAV	ssDNA/AuNPs	0.65 pM	10 fg μL^−1^ to 10 pg μL^−1^	91
HBV	Silver nanocluster-MoS_2_ nanosheet	10.7 nM	5–30 nM	92
HBV	Indium tin oxide nanowires	1 fM	1 fM to 10 μM	93
HCV	Carbon nanotube-cobalt NPs	8.82 × 10^−10^ M	1.0 nM to 12 μM	94
HIV	Antibody-graphene	100 fg mL^−1^	1 fg mL^−1^ to 1 μg mL^−1^	95
	AuNPs	0.1 pg mL^−1^	1000–0.1 pg mL^−1^	96
	Copper sulfide nanoplate	25 pM	0.05–1 nM	97

**Ebola**				
	ssDNA- AuNPs	4.7 nM	—	98
	GO- AuNPs	1 ng mL^−1^	1–400 ng mL^−1^	99

**Influenza**				
H1N1	Peptide-functionalized polydiacetylene	10^5^ PFU	—	100
H5N1	AuNPs	40–0.1 ng	100–0.1 ng	101
H5N1	Magnetic	1.0 nM	—	102

**Herpes**				
HSV-1	Carboxymethyl-dextran polymer sensor chips	5.2 × 10^−11^ M	5.2 × 10^−11^–1.3 × 10^−7^ M	103
KSHV	AuNPs	∼1 nM	1 Mm to 10 pM	104
HHV-5	Zinc–silver nanoblooms	97 copies per mL	113–10^3^ copies per mL	105

**Human papilloma**				
	Carbon nano-onions	0.5 nM	0.5–20 nM	106
	Au nanosheets	0.15 pM	1 pM to 1 μM	107
	Au nanotubes	1 fM	0.01 pM to 1 μM	108

A GE and single-strand DNA (ssDNA)-based electrochemical nano-biosensor can detect the Ebola virus.^98^ A nano-biosensor with outstanding chromatic features was fabricated by the nano-precipitation technique for H1N1 virus diagnosis. The change in color from blue to red is a very simple detection process. This nano-biosensor is suitable for transformation into commercial diagnostic tools for H1N1 virus detection.^100^ The human papillomavirus can also be diagnosed by DNA-based nano-biosensor composed of AuNPs. The electro-deposition approach has ensured the label-free detection of this sensor; AuNPs have been modified with nanoporous polycarbonate and utilized to develop this reproducible, quick, and stable sensor.^108^ Based on electrochemical and optical methods, nano-biosensors can detect HIV-1 and human T-cell lymphotropic virus-1 (HTLV-1) quickly, accurately, and economically in clinical therapy and control viral propagation.^129^ Some of the nano-biosensors, suitable for detecting other viral stains, including SARS-CoV, MERS-CoV, and human CoV, have been mentioned in Table 4.^109^

**Table 4:** A summary of nano-biosensors designed to detect COVID-19

Virus	Target	Method	LOD	Linear range	Specificity	Ref.
SARS-CoV	PP1ab gene	Chip-based colorimetric method by AuNPs	60 fmol	—	—	110
SARS-CoV	N-protein	Fluorescence fiber-optic biosensor	∼1 pg mL^−1^	0.1 pg mL^−1^ to 1 ng mL^−1^	—	111
SARS-CoV	N-protein	FET-based In_2_O_3_ nanowires	2–10 nM	—	High specificity but lack of statics	112
ARS-CoV	Thiolate-gene probe	Electrochemical method based on spherical AuNPs	3 pM	5–300 pM	0.463 μA pM^−1^	113
SARS-CoV	N-protein	AlGaN/GaN high electron mobility transistors	0.003 nM	0.4 pg	High specificity (33-fold larger than RT-PCR)	114
MERS-CoV	PNA probes	Paper-based colorimetric DNA sensor based on AgNPs	1.53 nM	20–1000 nM	High specificity but lack of statics	115
MERS-CoV	S-protein	Electrochemical method based on AuNPs	1.0 pg mL^−1^	0.01–10 000 ng mL^−1^	High selective	116
MERS-CoV	E-protein and open reading frames (ORF) gene	Colorimetric assays based on LSPR change	6 × 10^11^ copies per μL (1 pmol μL^−1^)	1.5 × 10^3^ to 6.7 × 10^3^ copies per μL	High specificity but lack of statics	117
Human CoV	S-protein	Electrochemical method based on AuNPs	0.4 pg mL^−1^	0.001–100 ng mL^−1^	High selectivity	116

### 2.7 Commercially available nano-biosensors

Examples of commercially available nano-biosensors are summarized in Table 5. These nano-biosensors are very fast in the detection of COVID-19. The detection time ranges from 10–30 min, which is faster and more convenient than the PCR test. No special or additional tools are required. Many of these nano-biosensors are highly specific (∼100%), reliable (∼100%), and sensitive (∼98%). The suitable storage temperature of these sensors is 2–35 °C, which is very simple and easy to maintain. Different variants of SARS-CoV-2, including Alpha (B.1.1.7), Beta (B.1.351), Gamma (P.1), Delta (B.1.617.2), Epsilon (B.1.429, B.1.427, CA.20C), Iota (B.1.526), Kappa (B.1.617.1), Mu (B.1.621), Fin-796H, and omicron (B.1.1.529) are detectable by these nano-biosensors. The nano-bisensors are user-friendly as all reagents are ready to use, and the result is visually readable.^118^ The rapid anti-HCV test is a commercially available nano-biosensor that is suitable for qualitatively detecting the hepatitis C virus (HCV) in humans. This tool is a colloidal gold enhanced rapid immunochromatographic assay for diagnosing the hepatitis C virus (HCV) antibodies in human blood, serum, and plasma.^119^ The sensor is highly sensitive (99.7%), specific (99.8%), and fast (15–20 min) in the detection of antibodies. The one-step Anti-HIV (1&2) test kit is another type of commercially available sensor for the qualitative detection of Human Immunodeficiency Virus (HIV) antibodies. The rapid flu A/B test is a rapid immunoassay for the qualitative detection of influenza A and influenza B viral nucleoprotein antigens in the human body. For Flu A, the sensitivity and specificity are 93.10% and 97.30%, respectively while for Flu B the sensitivity and specificity are 93.28% and 97.76%, respectively. Commercially available nano-biosensors for detecting SARS-CoV-2 (COVID-19) diagnosis are summarized in Table 5.

**Table 5:** A summary of the commercially available nano-biosensors for detecting SARS-CoV-2 (COVID-19)

Sl#	Producer	Brand name	Method	Technical details	Detection time	Sample type	Approval authority	Ref.
1	BioMedomics, Inc	COVID-19 IgM-IgG dual antibody rapid test	Qualitatively detect IgG and IgM antibodies	This test detects both early and late marker, of corona virus-associated IgM/IgG antibodies in human	10–15 min per test	Whole blood, serum, or plasma	EU (CE mark), USA (EUA)	120
2	Guangzhou wondfo biotech (Guangzhou, China)	Finecare SARSCoV-2 antibody test	Antibody test	Lateral flow immunoassay that detects IgM and IgG antibodies directed against SARS-CoV-2	15 min	Serum or plasma sample	National medical products administration EUA in China; CE mark in Europe	121
3	InTec PRODUCTS, INC.	Rapid SARS-CoV-2 antibody (IgM/IgG) test	IgM and IgG antibody detection	Apid immunoassay for the qualitative detection of IgM and IgG antibodies	NR	Single drop of fingerpick whole blood	EU, CE-IVD	122
4	Snibe diagnostic	MAGLUMI 2019-nCoV IgM/IgG (CLIA)	MAGLUMI 2019-nCoV IgM/IgG kit	Automated central laboratory rapid test that runs on MAGLUMI chemiluminescence immunoassay system	30 min	Blood sample	Automated IA, CEIVD	123
5	Sugentech	COVID-19 IgM/IgG	SGTi-flex COVID-19 IgM/IgG	SGTi-flex COVID-19 IgM/IgG	10 min	Human whole blood (finger prick or venous), serum or plasma	CE-IVD	124
6	VivaChek biotech (hangzhou) Co., Ltd	VivaDiag™	SARS-CoV-2 IgM/IgG rapid test (colloid gold)	*In vitro* qualitative assay to detects IgM and IgG antibodies against SARS-CoV-2	15 min	Whole blood (fingertip/venous), serum or plasma	CE-IVD	125
7	Quidel corporation	Sofia 2 SARS antigen FIA	SARS-CoV-2 antigen detection	Immunofluorescence-based lateral flow technology in a sandwich design for qualitative detection of nucleocapsid protein from SARS-CoV-2	15 min	Nasal swab, nasopharyngeal swab	FDA EUA	126
8	Avacta group plc	AffiDX^®^	SARS-CoV-2 antigen detection	Lateral flow test	20 min	Saliva, nasopharyngeal swabs or serum	CE (Europe), FDA (USA)	127
9	Coris bio concept	Affimer® biotherapeutics and reagents	COVID-19 antigen detection	COVID-19 antigen detection	NR	Saliva, nasopharyngeal swabs or serum		128
		CORIS COVID-19 Ag respi-stri	COVID-19 antigen detection	Immunochromatography assay based on a membrane technology with colloidal AuNPs fabricated with monoclonal antibodies directed against the conserved nucleoprotein antigen of SARS-CoV-2	15 min	Nasopharyngeal specimens	EU, CE-IVD	128

## 3 Selection and optimization of nanomaterials for nano-biosensors

It has been demonstrated that carbon nanoparticles do not cause any harmful threat to environmental components at lower concentrations. Carbon-assembly nanomaterials are especially nontoxic for micro-organisms and not very toxic to algae, fish, and other aquatic arthropods. Sustainable and low-cost carbonaceous materials like CNT and graphene are eco-friendly nanoparticles for designing and developing nano-biosensors.^130^

Different types of nanomaterials selected for manufacturing nano-biosensors are synthesized by following different approaches such as top-down or bottom-up methods. Carbon nanomaterials, including nanotubes, nanofibers, and graphenes (diameters ranging from 0.4 to 500 nm), are synthesized by the chemical vapor deposition (CVD) method from the decomposition of gaseous hydrocarbons over transition metal-catalyst particles.^131^ Other synthetic processes such as arc discharge, flame synthesis, and laser ablation are not suitable for bulk production since they are expensive, energy-intensive, and less efficient. Globally, many companies are manufacturing carbon nanoparticles. One of the most well-known suppliers is Pyrograf Products, Inc. (USA). Their prices vary from $85 per lb (electrical applications) to $170 per lb (thermal applications). Carbon NT&F 21, Catalyx Nanotech, Inc., Catalytic Materials LLC, CoMoCat®, *etc.*, are some of the famous carbon nanomaterial manufacturers that propose different prices for their products depending on the quality and purity. The selection and application of specific catalysts (*e.g.*, metals) can directly alter the chemical, physical, optical, electronic, and magnetic properties of such nanomaterials.

Inorganic nanomaterials such as metal nanomaterials (AuNPs, AgNPs, platinum nanoparticles (PtNPs), palladium nanoparticles (PdNPs), *etc.*), metal/carbon nanotubes hybrids, metal oxides (TiO_2_, ZnO, ZrO_2_, CuO, Al_2_O_3_, Fe_2_O_3_, Fe_3_O_4_, *etc.*) have the potentiality for the manufacture of nano-biosensors. The most common synthetic route for manufacturing AuNPs is by reducing a metal salt in a solution containing a stabilizer. The bulk production of AuNPs can be conducted by solvated metal atom dispersion technique (SMAD). The vaporization of Au leads to almost spherical AuNPs with sizes of 1 to 6 nm under a vacuum chamber in the presence of organic solvents. AgNPs can be produced by the reduction of a silver salt with a reducing agent (β-d-glucose, sodium borohydride, NaBH_4_) with the help of a colloidal stabilizer including polyvinyl alcohol (PVA), BSA, polyvinyl pyrrolidone (PVP), citrate, starch, and cellulose. PtNPs can be prepared by potassium hexachloroplatinate reduction in the presence of trisodium citrate and sodium dodecyl sulfate stabilizer under strenuous and continuous stirring. Pt-nanoflowers can be prepared by a simple and extensible method such as ethanol reduction at 85 °C with the help of PVP-10, a capping agent. The structure and order of Pt-nanoflowers can be precisely controlled by regulating reaction conditions and parameters. Bulk production of PdNPs can be achieved by the thermal decomposition of metal acetate solution in a surfactant at 300 °C. The monodispersity and particle size of PdNPs can be achieved by altering the surfactant molar ratio. PdNPs having an average size larger than 10 nm can be synthesized by applying oleylamine as a surfactant. The method is more advantageous than others because quality nanoparticles can be easily produced without the help of further selective techniques. Metal-carbon nanotube hybrids can be synthesized by spontaneous reduction, electrochemical deposition, physical evaporation, and solid-state reaction with metal salts at elevated temperatures. Metal oxide NPs are synthesized by vapor–liquid–solid (VLS) phase growth, solution–liquid–solid (SLS) phase growth, and vapor–solid (VS) phase growth processes without or with the help of a catalyst. Quantum dots (QDs) are colloidal semiconductor monodispersed nanocrystals with diameters ranging from 2–10 nm. They are synthesized by vapor phase reactions, hydrothermal and solvothermal techniques, ultrasound irradiation, reactions in confined solids, nanolithography, and microwave irradiation methods.

For the improvement of nanoparticles, pre-treatments or post-treatments are required. These modification procedures, including the infusion with metal-oxides, acid/base activations, and steam/CO_2_ activation, have their limitations, such as being complicated and slow, requiring supporting processes, and having high operation costs. The functionalization of nanomaterials can be performed by several techniques including physical, chemical, and biological modifications. Treatments with steam/gas, microwave, and magnetic forces are considered as physical modifications, while chemical modifications are done with H_2_O_2_, HCl, H_2_SO_4_, HNO_3_, H_3_PO_4_, KOH, NaOH, impregnation, and coating. Both the physical and chemical modification methods have advantages and disadvantages. Steam or gas treatment is suitable for producing commercially bulk amounts of engineered nanomaterial with improved porosity, surface area, and surface reactivity. However, this process is difficult to control. On the contrary, the microwave technique is easy to control, cost-effective, and more functional for producing nanomaterials with more functional groups and higher surface area, but the process is energy-intensive. For the removal of surface contaminants, the magnetic separation method is applicable, but the magnetic attraction impacts the particle's surface area. Treatment with H_2_O_2_ is cheap and eco-friendly, and yields improved nanoparticles with oxygen-containing surface area but the yield is very low. The treatments with acids and alkali yield particles with higher carboxyl functional group-containing surfaces but pollute the environment. For improved surface area, anion–cation exchangeability, negative surface charge density, and hydrophobicity of nanomaterials, biological modifications such as bacterial conversion and anaerobic digestion are effectively applied.

The price of 1 mg AuNPs varies from US$20 to US$40, while 1 mg of PVP stabilized AuNPs costs US$180 and functionalized AuNPs cost US$250. PtNPs are the most expensive; 1 g costs US$2500. PdNPs are also very expensive; for example, 1 g costs US$1400. Among the nanomaterials, the price of QDs is very high, which varies from US$2000 to US$10,000 for 1 g. The price of nanoparticles is still very high. It needs extensive research to minimize the production cost by reassessing several parameters, including energy consumption for nanomaterial synthesis and the construction of equipment and facilities. Conventional waste management strategies should be modernized to determine nano-ecotoxicology. Since the demand for nanomaterials is increasing rapidly, large-scale industrial production is essential. As such, the pros and cons of laboratory and synthetic industrial processes should be analyzed for adopting cost-effective and eco-friendly techniques for the bulk and mass production of nanomaterials.

The eco-friendly operating conditions (optimum process temperature, solvent-free or environmentally benign solvent and auxiliaries), high reaction selectivity, utilization of non-toxic catalysts and safer chemicals, higher atom economy, renewable feedstock, energy-efficient operating conditions, short reaction time, selection and designing of biodegradable raw materials and finished products, real-time analysis for pollution prevention, and the selection and utilization of inherently safer chemicals may help the manufacturer to adopt an overall sustainable technique for the selection and optimization of nanomaterials for developing nano-biosensors.

## 4 Current challenges and prospective solutions

Like other technology, nanotechnology is comprised of many technical disciplines, including but not limited to physics, chemistry, biology, material science, electronics, and self-assembly, and has certain drawbacks associated with nanomaterials or nanoparticles involved in the design and development of nanotechnology products. Due to the extraordinary physicochemical characteristics of nanoparticles, their toxicological and hazardous effects on the environment and health are entirely different as compared to those imposed by the same bulk materials. In addition, nanoparticles decompose very slowly, so there is a high probability of their accumulation at the area of administration. Nanoparticles undergo various aging processes in the environment and living bodies, leading to eco-toxicity and the damage of organs. This challenge can be addressed by utilizing biodegradable and safer nanoparticles such as clay, cellulose nanomaterials, halloysite nanotubes, starch nanocrystals, nanocarbon (graphene, graphene nanoplatelets, graphite, *etc.*), and carbonaceous nanomaterials (carbon nanotube, nanospheres, *etc.*).

One of the major challenges in COVID-19 diagnosis is the nano-biosensors’ lack of high selectivity and specificity, which escalate the error in the detection process of samples due to the mutations of virus proteins and mRNA. According to the reports, it seems that the simultaneous use of several diagnostic factors such as opening reading frame 1a/b (ORF1a/b), RNA-dependent RNA polymerase (RdRp) genes, and so on, with nucleocapsid and spike proteins (N- and S-proteins) can remarkably enhance the specificity and selectivity of the sensors.^109^ The origin of the analyzed samples influences consequential ambiguities in the susceptibility and particularity of the nano-biosensors for COVID-19 detection. Reaching the origin of contamination in olfactory ducts, larynx, and mouth can improve the susceptibility and particularity of COVID-19 detection. Nevertheless, blood samples are employed to identify Ig for COVID-19 detection. Hence, the utilization of blood samples in the COVID-19 diagnosis appears to be more pertinent due to the approach to all the aspects of COVID-19. The unavailability of an appropriate platform for determining viruses is one of the major challenges in designing and developing nano-biosensors. Significant improvements in smartphone technology can provide a platform for detecting viruses quickly and easily through a cost-effective process. Due to the global widespread presence of COVID-19, the layout and fabrication of simple nano-biosensors with improved performance and precision, like blood sugar sensors, is crucial. Nonetheless, nano-biosensors manufactured in the diagnosis of COVID-19 are multiplexed and require complex sample preparation. As such, a prime challenge is the manufacture and distribution of a straightforward nano-biosensor with high susceptibility and particularity for mass access. A better interpretation of the molecular mechanism and the interactions of all the components present in a nano-biosensor and a clear understanding of their molecular structures would be a major breakthrough to address this prime challenge. In the future, the effective application of machine learning algorithms and artificial intelligence may enhance the accuracy and precision of the nano-biosensor for COVID-19 detection. Large-scale clinical trials and complex sample processing techniques are crucial for understanding sensor stability and commercial feasibility.

## 5 Ethical and technological considerations for the adoption of nano-biosensors

The COVID-19 pandemic has had an enormous global impact. In the present situation, it is essential to extend our attention to the ethical issues that were and still are uplifted during this COVID-19 crisis and in managing the future wave. Ethical issues regarding testing and tracing of COVID-19 patients need to be highlighted, focused on, and discussed. In association with diagnosis, there are questions about whether it is ethical to utilize the detection techniques with low reliability for increasing the diagnostic capacity for reducing harm for COVID-19 patients. Another issue is how vast and selective the testing should be and whether selective testing is in conformity with the moralities of social justice. People infected and recovered from COVID-19 might have some immunity; however, ascribing defined ‘immuno-privileges’ raises some ethical issues. Applying different tracking strategies, including apps, databases, tracers, *etc.*, has raised ethical questions of social justice and privacy.^132^ Specific ethical issues are related to several categories of persons, including persons not known to have been exposed to or infected with SARS-CoV-2, persons known to have been exposed to the virus, persons suspected to be infected, persons who are known to be infected, and persons who are known to have recovered from COVID-19 and are adequately immune. In the case of persons suspected to be infected, one ethical issue is selecting the person who should be tested and which types of tests are justified in a place where tests are insufficient as compared with demand. From a clinical ethical point of view, it is reasonable to choose the test that has the highest sensitivity and selectivity since a COVID-19 diagnosis has further repercussions, including isolation and being treated separately on a COVID-19-dedicated ward. Due to these consequences, it is essential to reduce errors such as false negatives or false positives.

According to the clinical diagnostic point of view, tests conducted should be reliable, but the test does not have to be cost-effective. However, from a public health standpoint, it is essential to expand the range of test capabilities that can be accomplished quickly. Simultaneously, the test should be cost-effective and more reliable with higher selectivity and sensitivity. To achieve standard management for the COVID-19 pandemic, it is necessary to balance clinical and public health perspectives. The RT-PCR is a gold standard test for COVID-19 detection. A test with lower analytic susceptibility but easily and frequently applicable could be economical. To combat the fast spread of the virus, a selection must be made to redistribute test facilities or apply inexpensive tests for COVID-19 detection. There is no question that we are facing a global catastrophe but this does not mean that our basic rights are automatically relinquished. Keeping the clinical ethical issues balanced, public health ethics should be justified simultaneously. There is no doubt that the RT-PCR test is the most selective and sensitive diagnostic tool, which is why it has become the most important, making it the most helpful from a clinical point of view. However, during a public health emergency, it is necessary to contemplate the best way to distribute tests. In this attempt, it is acceptable to conduct tests that are less sensitive but more quickly available at a low price.

Technology adoption of nano-biosensors is limited by a multidimensional technology assessment process concentrated on the outcomes. The limitations of technology adoption can be vanquished through mediator activities such as product certification, extensive technology assessments, and effective real-life applications.^133^ The associated sectors are changed quickly in response to technological innovations, while demand plays a vital role in accepting new technologies. Technology adoption is a sequential process influenced by technical and social governance factors. Comprehensive exploratory strategies, future research, sustainable design, more quantitative forms of modeling, a critical transformation of the lab manufacturing process into mass production, and the broader yet inter-connected decision of policy-makers may facilitate the technical adoption of nano-biosensors. Adaptation of new technology always requires substantial time that allows technological advancement and social acceptance throughout the development of a harmonic strategy to achieve academic research, industrial production, and societal goals. The design and development of stable, reliable, fast, cheap, simple, specific, and effective nano-biosensors for COVID-19 diagnosis may take some more time to be adopted technically. However, during this emergency, the required time for technology adoption may be reduced for pandemic management.^134^

## 6 Future perspectives and conclusions

The whole world is bedeviled by the jeopardy of COVID-19. Both the treatment and detection of COVID-19 remain challenging even though scientists have succeeded in understanding, interpreting, and identifying many facts about the virus. Accurate diagnosis is the first stride to begin the treatment of COVID-19 patients. The conventional procedures adopted for SARS-CoV2 detection are still unable to serve as mass screening techniques. Hence, designing more reliable, rapid, specific, inexpensive, handy, simple, and widely accessible diagnostic tools should be introduced and commercialized. Some advanced nanotechnology approaches based on fluorescent markers, electrochemical responses, colorimetric detection, and magnetic field-induced identifications have been highlighted in this review. These innovative tools have increased the daily number of tests qualitatively and quantitatively. But the nano-biosensors capable of detecting considerably lower concentrations with high specificity in the presence of similar contaminants or analysts are still challenging. The current nano-biosensor research should thus be focused on having the sensor with greater accuracy, faster response time, minimal post-processing steps, capable of regeneration for reuse, longer life, and cost-effectiveness. The binding affinity and specificity of the nano-biosensors for the SARS COV-2 virus must be further developed for use as diagnostic devices to manage unforeseen medical emergencies.

## Conflicts of interest

There are no conflicts to declare.

## Supplementary Material
